# A Report on the Influence of Climate on Pulmonary Diseases in Minnesota

**Published:** 1877-03

**Authors:** 


A REPORT ON THE INFLUENCE OF CLIMATE ON PULMINARY DISEASES IN MINNESOTA. By Franklin Staples, M.D. Minnesota Extracts from the Transactions of the American Medical Association.

This is a statistical record showing the benefit to pulmonary troubles from the salubrious climate of Minnesota. This pathological condition is adapted to the climatic benefits of the State, as proven by the opinions and experience of resident physicians.

While phthisis pulmonalis is not altogether absent, it is found to result principally from acute pneumonia and does not invalidate the theory of the beneficial influence of the climate upon idiopathic tuberculosis.

The zeal of the profession of Minnesota is commendable, and cannot fail to awaken, both by its precepts and example, a lively interest in the profession at large in all medical investigations of this subject.

